# A Two-Stage, Intelligent Bearing-Fault-Diagnosis Method Using Order-Tracking and a One-Dimensional Convolutional Neural Network with Variable Speeds

**DOI:** 10.3390/s21030675

**Published:** 2021-01-20

**Authors:** Mengyu Ji, Gaoliang Peng, Jun He, Shaohui Liu, Zhao Chen, Sijue Li

**Affiliations:** 1 State Key Laboratory of Robotic Technology and System, Harbin Institute of Technology, Harbin 150000, China; 19B308008@stu.hit.edu.cn (M.J.); hejun_hit@126.com (J.H.); 17766589892@163.com (Z.C.); lisijue@hit.edu.cn (S.L.); 2 Harbin Institute of Technology, School of Computer Science and Technology, Harbin 150000, China; shliu@hit.edu.cn

**Keywords:** bearing fault diagnosis, variable speeds, order tracking, one-dimensional convolutional neural network

## Abstract

When performing fault diagnosis tasks on bearings, the change of any bearing’s rotation speed will cause the frequency spectrum of bearing fault characteristics to be blurred. This makes it difficult to extract stable fault features based on manual or intelligent methods, resulting in a decrease in diagnostic accuracy. In this paper, a two-stage, intelligent fault diagnosis method (order-tracking one-dimensional convolutional neural network, OT-1DCNN) is proposed to deal with the problem of fault diagnosis under variable speed conditions. Firstly, the order tracking algorithm is used to resample the monitoring data obtained under different rotation speeds. Then, the one-dimensional convolutional neural network is adopted to extract features of the fault data. Finally, the fault type of collected data can be obtained by fully connected networks based on the features extracted. In the time domain, while the proposed algorithm only relies on the fault data collected under one speed as the training dataset, it is capable of doing fault diagnosis under different speed conditions. In the condition with the largest difference in speed with each dataset, the accuracy of the proposed method is higher than the baseline methods by 0.54% and 11.00%—on CWRU dataset and our own dataset respectively. The results show that the proposed method performs well in dealing with the fault diagnosis under the condition of variable speeds.

## 1. Introduction

With the development of science and technology, mechanical equipment is becoming more and more automatic and intelligent. As the key parts of mechanical equipment, rotating mechanical parts such as bearings and lead screws play an important role in the overall performance of the equipment [1]. When the bearing parts are damaged and fail, the precision of the equipment will decline rapidly; eventually there will be equipment failure and casualties. Therefore, it is very necessary to monitor the working state of the bearing parts [2].

Bearing fault diagnosis is a hot research field of mechanical condition monitoring. The extraction of monitoring signals’ features and pattern classification are the core steps of bearing fault diagnosis. Among various kinds of monitoring signals, the vibration signal, which has the advantages of being easy to monitor and rich in information, is widely used in the field of mechanical condition monitoring. When a certain part of the bearing fails (such as cracking of the rolling body and fatigue pitting corrosion of the inner ring or outer ring rolling), the bearings will produce periodic additional vibrations. The frequency of the additional vibration has a certain relationship with the bearing speed (Equations (2)–(5)), which is called the fault characteristic frequency. When transforming the vibration signal to the frequency domain, the signal components with the fault characteristic frequency will have a large amplitude. By identifying the frequency components of the original vibration signal, we can identify where the bearing failed.

For the bearing fault diagnosis based on the vibration signal, the common feature extraction methods based on classical signal processing methods mainly include the Hilbert–Huang transform (HHT), the wavelet transform, empirical mode decomposition and methods based on largest Lyapunov. V.K. Rai et al. [3] adopted HHT to extract the frequency domain characteristics of the bearing fault data, realizing the purpose of identifying the bearing fault types. Xinsheng Lou et al. [4] proposed a new scheme based on wavelet transform and neuro-fuzzy classification for ball bearing fault diagnosis. This method used the wavelet transform to extract the feature vectors of the accelerometer signals. Then the adaptive neural-fuzzy inference system was trained to classify the feature vectors. The proposed method performed well under the variable load conditions. Yang Yu et al. [5] proposed roller bearing fault diagnosis based on EMD energy entropy and an artificial neural network (ANN). Intrinsic mode functions (IMFs) are extracted from the original acceleration vibration signals. Then energy features extracted from the IMFs are sent into the ANN; the fault patterns can be identified eventually. Wahyu Caesarendra et al. [6] applied the largest Lyapunov exponent (LLE) algorithm in low speed slew bearing condition monitoring. The method is able to detect the change in the state of slew bearing and performs better than the comparable methods.

In recent years, the intelligent fault diagnosis methods based on machine learning and deep learning have achieved excellent results in the field of bearing fault diagnosis. P. Konar et al. [7] successfully applied the SVMs in the field of fault diagnosis. They adopted a continuous wavelet transform (CWT) to extract the feature vectors, and then a support vector machine (SVM) was used to classify the monitoring data of the three-phase induction motor. Zhuanzhe Zhao at al. [8] proposed an intelligent fault diagnosis method based on a back propagation (BP) neural network to recognition the early fault of the bearing. The proposed method firstly used a wavelet packet decomposition method for de-noising, and then the intrinsic mode functions (IMFs) were obtained with the EMD method. Finally, a three-layer BP neural network was established to identify the fault pattern of the monitoring signals. V. Muralidharan et al. [9] adopted a naïve Bayes classifier and Bayes net classifier to perform the task for fault diagnosis. The proposed method firstly extracts the discrete wavelet features of the vibration signals by wavelet analysis; then the features are used as the input of the Bayes net for classification. In the literature [10], they also attempted to conduct the bearing-fault-diagnosis task with the method of Hilbert–Huang transform (HHT) and the K-nearest neighbors algorithm. Among the above methods, the classical signal processing algorithms are used to manually extract the feature vectors in the original signals. Then, the methods based on machine learning are adopted to classify the signals according to the extracted feature vectors.

Deep learning methods such as convolutional neural network can automatically extract the high dimensional and low dimensional information in the original vibration signal. After network training, fault-related feature information in the original vibration signals can be effectively extracted and enhanced, and then the vibration signal can be classified. In our previous work [2], a one-dimensional convolution neural network was proposed to accomplish the task for bearing fault diagnosis. In this work, the steps of extracting feature and pattern recognition were completed through convolutional layers and fully connected layers automatically. To overcome the problem of gradient vanishing or exploding in the network training process, Xiang Li et al. [11] proposed a deep residual learning-based fault diagnosis method for machinery. This method is proven to be able to improve the information flow throughout the network. Reference [12] adopted the recurrent neural network to detect the real-time running state of the gas turbine engines. By approximating the probability distribution of the monitoring signals, the network is used to determine whether the equipment is in normal state. Reference [13] adopted unsupervised learning to detect the working state of a gas turbine. The proposed method used an auto-encoder with additional weight to extract the temperature curve. It gets rid of the dependence on the prior dataset and has great academic and practical value. The methods based on the modified convolutional neural networks have made great strides in the field of fault diagnosis. However, the existing problem is that they require the training datasets and the testing datasets to follow the same distribution. However, when the speed of the bearing is unstable, the distribution of fault data will also change, despite the fact that the fault type of the bearing is the same. In this condition, the accuracy of the diagnosis networks will drop dramatically. To solve this problem, we must adopt the necessary preprocessing methods to make the data follow the same distribution.

Compared with the benign situation that the bearing rotates at constant speed, this paper addresses a much more challenging problem where the bearing has variable speed, which directly leads to changes in the distribution of vibration signal, and hence makes it more difficult to diagnose. This challenge has been widely recognized in the bearing fault diagnosis community and still remains an open problem.

The methods based on time-frequency domain analysis [14] and transfer learning [15,16] perform relatively well in dealing with the fault diagnosis problems under the conditions of variable speeds and different equipment. Among the existing fault diagnosis methods, the order tracking technology is the most direct and effective method to deal with the bearing fault diagnosis under the condition of variable speed [17]. Order tracking adopts different sampling frequency to collect the fault signal according to the different rotating frequency of the bearing. It can convert the non-stationary time-domain signal into an angular domain signal for analysis. That way, order tracking can overcome the problem of different data distributions caused by the variable speed condition. However, the process of fault diagnosis based on order tracking mostly needs manual analysis, which is complicated and has a low degree of automation. This limits its practical application in the industrial production process. The methods based on transfer learning have the characteristics of intelligence and automation. They can deal with the difference in data distribution of the source domain and target domain. However, most of these methods focus on adapting to diagnosis tasks between the different equipment, and less attention is paid to adapting to tasks under the condition of variable speeds. Additionally, the effect of those methods in the multi-distribution domains transfer task for bearing fault diagnosis is not ideal.

To sum up, the methods of bearing fault diagnosis can be divided into four categories: methods based on classical signal processing algorithms in time domain/frequency domain/time-frequency domain analysis, methods based on traditional machine learning, methods based on deep learning and methods based on transfer learning [18]. These methods have their own advantages and disadvantages in dealing with the bearing fault diagnosis in different scenarios and working conditions. However, the current research on fault diagnosis is mainly focused on the condition with the single rotating speed. These methods are not suitable for the diagnosis under the condition of variable speeds.

This paper proposes a novel, intelligent fault diagnosis method for bearing fault diagnosis under variable speed conditions. Firstly, the order tracking algorithm is adopted for speed normalization of fault data under variable speed conditions, and then the one-dimensional convolutional neural network is used to extract the fault feature vectors and classify the fault data automatically. The proposed method takes advantage of the order tracking and deep convolutional neural network; the former resamples the fault data collected at variable speed; the latter extracts the characteristics of the resampled data and classifies the resampled data automatically. By using different data processing methods at different stages, the migration diagnosis from the fault data collected at a certain speed to the fault data collected at other speeds is finally realized.

The contributions of this paper are as follows: Firstly, the proposed method is based on the convolutional neural network structure, and we develop the 2D-CNN into a 1D-CNN. The proposed 1D-CNN can adaptively extract the features of the monitoring data. This avoids the complex operations of manually designing and extracting the features of the fault data. Secondly, the proposed method has the least decrease in accuracy compared with the comparative methods dealing with the monitoring data collected at variable speeds. As OT-1DCNN adopts the order tracking algorithm to preprocess the original vibration signal. It can fundamentally solve the problem of changing the frequency spectrum characteristics of the fault data caused by variable speed conditions. Finally, the proposed method reduces the requirements for the completeness of the training datasets. The method only needs the data collected at just one speed, as the training dataset and the data with different distributions collected at other speeds can be classified at the same time. Additionally, the network does not need to be retrained. Differently from the intelligent diagnosis methods, the proposed network neither includes the domain adaptive network, nor needs to design and extract the distribution difference metric between different domain data. The structure and training process of the neural network are very simple.

The rest of this paper is organized as follows: In Section 2, we introduce the technical background and the details of the proposed algorithm. This section consists of three parts. In the first part, we give a brief review of the order tracking. Additionally, in the second part, the relevant knowledge of DCNN is introduced. Finally, we describe the proposed two-stage, intelligent fault diagnosis method in detail. In Section 3, we validate the performance of the proposed algorithm with the extensive experiments on the CWRU bearing dataset and our own dataset respectively. In Section 4, the advantages and future works of the proposed method are discussed. The conclusions are drawn in Section 5.

## 2. Materials and Methods

### 2.1. A Brief Review of the Fault Diagnosis Based on Order Tracking at Variable Speed

**Figure 1:**
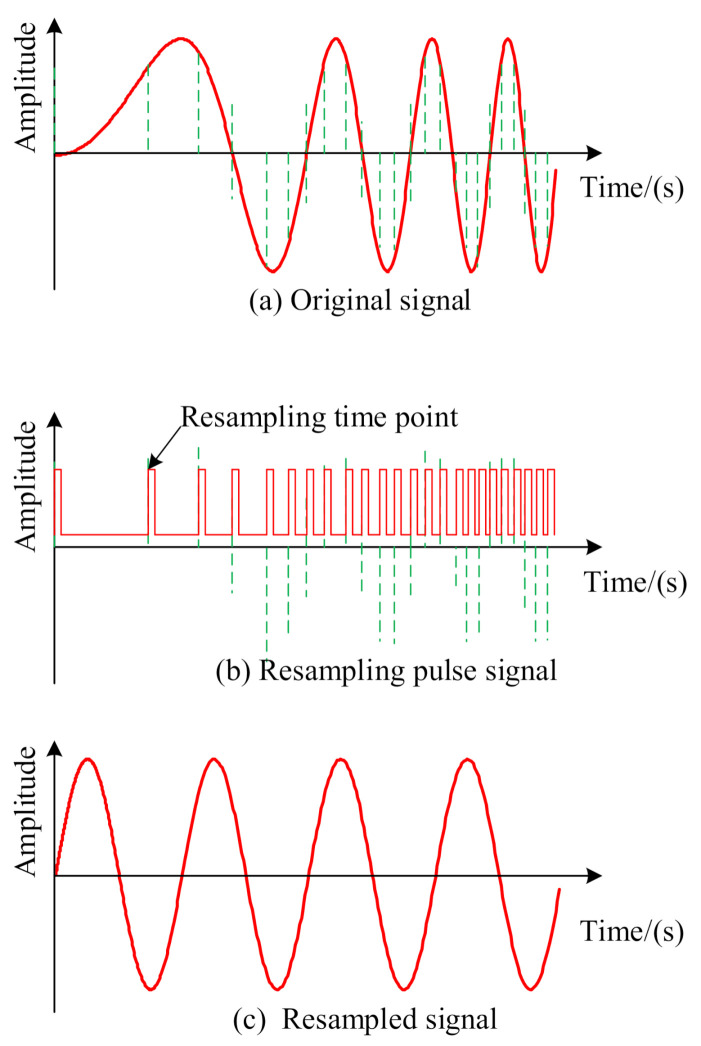
Schematic diagram of order tracking.

As the most effective method to deal with the fault diagnosis under the condition of variable speeds [19,20,21], the order tracking technique has been paid much attention by researchers. The essence of order tracking is to convert the non-stationary time-domain signal into a stationary angular domain signal. To achieve this, different sampling frequencies are adopted for monitoring signals at different rotating speeds, which is shown in Figure 1. By doing this, the problem of different distribution of the same fault type at variable speeds will be overcome [14].

In 2006, Saavedra P.N. [20] put forward the concept of order tracking for the first time; he introduced and analyzed the detailed process of order tracking. Order tracking technology can be roughly divided into four categories: hardware order tracking technology, computed order tracking technology, tacholess order tracking and Vold–Kalman order tracking [22,23]. The hardware order tracking algorithm controls the sampling frequency by using devices such as a key-phase device, a sampling rate synthesizer and a carrying aliasing tracking filter [19,24,25]. With this method, the stable signal in the angle domain can be obtained directly. The hardware order tracking method has great real-time performance and accuracy, but sometimes the cost of hardware is very high.

Hardware order tracking technology faces the difficulties of complex equipment and high testing costs in practical applications [26]. Bossley K.M. et al. [27] proposed the computed order tracking technology. It synchronously samples the vibration signals and the speed signals of the rotating machinery; calculates the phase time marker with the speed pulse signals; and then uses the interpolation algorithm to resample the original vibration signals [20,27]. Compared with the hardware order tracking algorithm, the requirement of computed order tracking algorithm on hardware equipment is greatly reduced.

Tacholess order tracking is suitable for cases where the instantaneous speeds are difficult to obtain. When limited by the structure space and hardware cost of the equipment, there may be no speed sensors in the monitored equipment, so the speed information cannot be obtained directly. The tacholess order tracking method attempts to extract the speed signals directly from the vibration signals by some methods, and resamples the vibration signals in the angle domain according to the extracted instantaneous speed signals [28,29,30]. Although this method can deal with the problem of spectral ambiguity caused by the fluctuation of rotation speeds, the method based on numerical integration will cause the deviation of the estimation of rotational speed phase. This will have a certain impact on the accuracy of the estimation of rotational speeds, which eventually leads to a decrease in accuracy of fault diagnosis.

Vold–Kalman order tracking focuses on how to extract the signal components of each order from the original vibration signals, and then analyzes the time domain waveform of each order component [31,32,33,34]. The Vold–Kalman order tracking method has low computational efficiency and is more suitable for offline diagnosis, which makes it difficult to make online diagnoses.

As can be seen from the above literature review, the order tracking algorithm is a powerful tool to deal with the problem of fault diagnosis under variable speed conditions. It can essentially overcome the influence of variable speed conditions on the distribution of fault data.

### 2.2. A Brief Review of the Fault Diagnosis Based on Deep Learning Methods under the Variable Speed Condition

In recent years, with the development of deep learning technology, the fault diagnosis algorithms based on neural networks have been used to solve the migration diagnosis problem under the condition of variable speeds. Viet Tra et al. [35] adopted the CNN network trained by the stochastic diagonal Levenberg–Marquardt algorithm to carry out migration diagnosis of bearing faults at variable speed conditions. This diagnostic method firstly performs a fast Fourier transform on acoustic emission signals and generates the spectral energy maps, and then extracts the fault feature and classifies the spectral energy maps using CNN network. The study [36] added the nuisance attribute projection (NAP) to the loss function of the CNN, and then trained the CNN with the fault data at several speeds. NAP is usually used in speed recognition; it was used to map the original fault signal to the feature domain to eliminate the influences of load, rotation speed and the noise. The CNN after training was used to classify the fault data collected at various rotating speeds.

In order to solve the problem of fault diagnosis for heterogeneous data collected under variable working conditions and variable equipment, the transfer learning method was introduced into the field of fault diagnosis. Kun X et al. [37] proposed a multilayer transfer convolutional neural network (MTCNN) for the diagnosis of bearing faults under variable speed conditions. This method uses a CNN network to extract fault data features. Then, the MMD method is used in the full connection layer to measure the distribution distance of fault data at different rotating speeds. Finally, the migration network is used to complete the migration diagnosis task. To further deal with the problems under the condition of variable speeds, they also proposed a renewable fusion fault diagnosis network (RFFDN) [38]. The model was applied to solve the problem in the case of sampling data missing at variable speeds. The network could update itself as the sample data came in.

The above intelligent fault diagnosis methods based on deep learning have achieved certain success in dealing with the problem of fault diagnosis at variable speeds. However, their training data sets all need to contain the fault data collected at two or more speeds, which significantly increases the difficulty and cost of making the training dataset. Few intelligent fault diagnosis methods based on deep learning only use the training datasets collected just under a single rotating speed condition. This is the significant disadvantage of existing intelligent fault diagnosis algorithms.

### 2.3. The Proposed Two-Stage, Intelligent Fault Diagnosis Algorithm

#### 2.3.1. Order-Tracking One-Dimensional Convolutional Neural Network

As is mentioned above, order tracking is superior in the ability to map the unstable time domain signal to the angular. It can essentially overcome the impact of variable speed operating conditions. For the monitored one-dimensional vibration signal, the proposed 1-DCNN convolutional neural network uses a one-dimensional wide convolution kernels (convolution kernel size is 64) in the first convolution layer. With the wide convolution kernels, the first layer in the network can obtain the frequency domain information in the signals. The proposed network uses the small convolution kernels in the subsequent layers to obtain more detailed features. 1-DCNN has the ability to automatically extract the weak fault features from the vibration data. Therefore, the combination of these two methods might be a robust approach.

**Figure 2:**
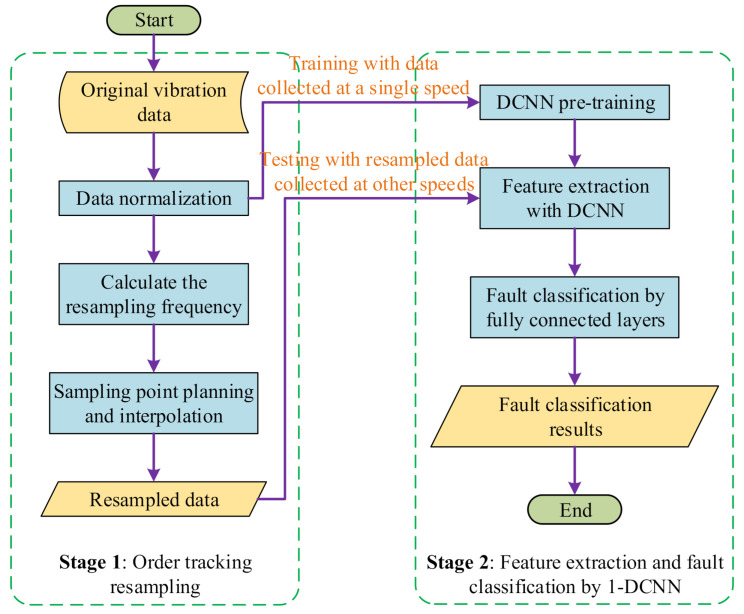
The flow chart of the proposed two-stage, intelligent diagnosis algorithm.

This paper proposes a two-stage, intelligent fault diagnosis algorithm to deal with the problem of fault diagnosis under variable speed conditions. Differently from the current fault diagnosis methods for variable speed working conditions, the proposed algorithm reduces the requirements on the data set. It only relies on fault data collected at one speed to train the network. After training, the proposed network has the ability to classify the resampled data collected at other speeds. In detail, the proposed algorithm firstly uses the order tracking algorithm to resample the original vibration signals collected at other speeds. After that, the resampled signals are obtained. Then, the resampled signals are sent to the pre-trained network. Finally, we can get the classification results of the fault data. The proposed method adopts the order tracking algorithm and a deep convolutional neural network in different processing stages, relies on the fault data collected at one speed as the training dataset and realizes the migration fault diagnosis of the fault data obtained under the other speed conditions. As a whole, the proposed algorithm finally uses the intelligent migration fault diagnosis from the fault data at a single speed to judge the fault data collected at other speeds. The flow chart of the proposed two-stage method is shown in Figure 2.

The proposed two-stage, intelligent fault diagnosis algorithm not only adopts the order tracking method to deal with the variable speed conditions, but also has the ability to extract the feature vectors of the fault data automatically. In the time domain, the proposed algorithm only relies on the fault data collected at one speed as the training dataset, and realizes the migration fault diagnosis of the fault data obtained under the other speed conditions.

#### 2.3.2. The Computed Order Tracking Algorithm

In the field of bearing fault diagnosis, the order is defined as the ratio of the fault characteristic frequency to the bearing speed [39]. It can be expressed by the following formula:

(1) $$O=\frac{f_{fault}}{f_{r}}=\frac{60f_{fault}}{n_{r}}$$

where *O* represents the order of fault; $f_{fault}$, $f_{r}$, $n_{r}$ are the fault frequency, bearing rotation frequency and bearing speed respectively.

For rolling bearings, the common failure types include outer ring failure, inner ring failure, rolling element failure and cage failure. The fault characteristic frequencies of various fault types are expressed as follows:

(2) $$f_{o}=\frac{1}{2}f_{r}\left(1-\frac{d}{D}\cos\alpha \right)Z$$

(3) $$f_{i}=\frac{1}{2}f_{r}\left(1+\frac{d}{D}\cos\alpha \right)Z$$

(4) $$f_{b}=\frac{1}{2}\frac{D}{d}f_{r}\left[1-{\left(\frac{d}{D}\right)}^{2}\cos^{2}\alpha \right]$$

(5) $$f_{c}=\frac{1}{2}f_{r}\left(1-\frac{d}{D}\cos\alpha \right)$$

where $f_{o}$, $f_{i}$, $f_{b}$, $f_{c}$ respectively represent the characteristic frequency of outer ring failure, the characteristic frequency of inner ring failure, the characteristic frequency of rolling element failure and the characteristic frequency of cage failure; d, D are the diameter of the rolling elements and the pitch diameter respectively; Z is the number of the rolling elements and α is the contact angle.

It can be seen from Equations (2)–(5) that when the structural parameters of the bearing are fixed, the frequency of each type of fault is linearly related to the frequency of rotation. When the speed of the bearing changes, the characteristic frequency of the bearing fault will also change.

Correspondingly, we can get the order of various faults as follows:

(6) $$O_{o}=\frac{1}{2}f_{r}\left(1-\frac{d}{D}\cos\alpha \right)Z$$

(7) $$O_{i}=\frac{1}{2}\left(1+\frac{d}{D}\cos\alpha \right)Z$$

(8) $$O_{b}=\frac{1}{2}\frac{D}{d}\left[1-{\left(\frac{d}{D}\right)}^{2}\cos^{2}\alpha \right]$$

(9) $$O_{c}=\frac{1}{2}\left(1-\frac{d}{D}\cos\alpha \right)Z$$

where $O_{o}$, $O_{i}$, $O_{b}$, $O_{c}$ are the outer ring fault order, inner ring fault order, rotating body fault order and cage fault order respectively.

It can be seen from Equations (6)–(9) that when the structural parameters of the bearing are fixed, the characteristic orders of the bearing faults are also fixed. The characteristic orders of the bearing faults do not change along with the variable speeds. This characteristic of fault feature orders provides strong theoretical support to deal with the problem of fault diagnosis under variable speed conditions.

**Figure 3:**
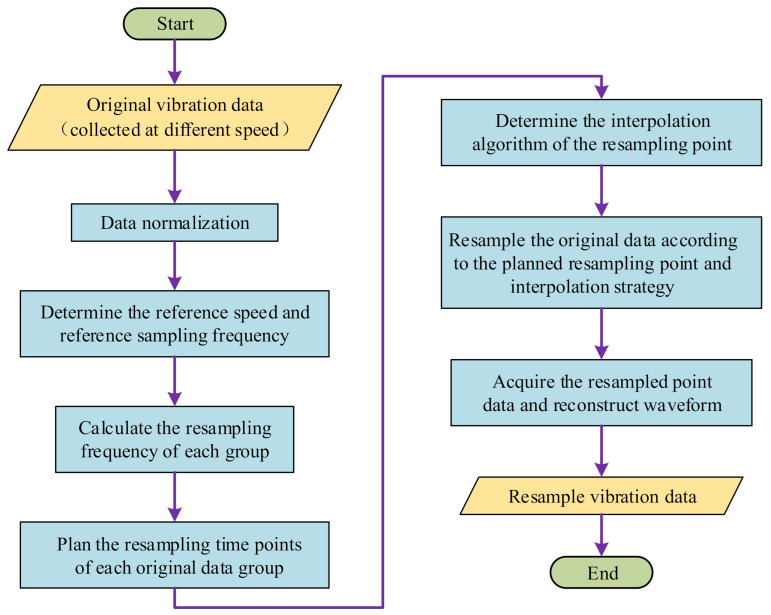
The implementation flow of the order tracking algorithm.

In actual industrial scenarios, the bearing has many working conditions that are fixed at several certain speeds. For such cases, the implementation flow of the order tracking algorithm is shown in the Figure 3.

The detailed steps of the order tracking algorithm are described as follows:

Monitor the fault response of the monitored bearing under different speeds; obtain the fault response data x of the bearing at a fixed sampling frequency $f_{s}$. According to the different rotating speeds $f_{ri}$ (i=1,2,⋯,n) of the bearing, divide the original monitoring data x into different fragments $x_{i}$(i=1,2,⋯,n).

Determine the reference speed $f_{base}$. In order to ensure the accuracy of the resampled data, we usually take the minimum speed of the typical working speed $f_{ri}$ of the bearing as a reference speed $f_{base}$, which is calculated as follows:

(10) $$f_{base}=\min\left(f_{r1},f_{r2},\cdots ,f_{rn}\right)$$

The original sampling frequency at the reference speed is the basic sampling frequency for resampling, which is calculated as follows:

(11) $$RF_{base}=f_{s}$$

Calculate the resampling frequency $RF_{i}$ of the monitoring data fragments at each speed, which is calculated as follows:

(12) $$RF_{i}=RF_{base}\frac{f_{ri}}{f_{base}}$$

Plan the resampling time $t_{rij}$(i=1,2,⋯,n, $j=1,2,\cdots ,p_{i}$) of the resampling points in each $x_{i}$. Firstly, assign the original sampling time points $t_{ij}$(i=1,2,⋯,n, $j=1,2,\cdots ,m_{i}$) to each original vibration data sampling point. Secondly, calculate the resampling time points $t_{rij}$ according to the re-sampling frequency $RF_{i}$. Finally, search the monitoring data values in the original sampling data sequence $x_{i}$ according to $t_{rij}$. $t_{ij}$ and $t_{rij}$ are calculated as follows:

(13) $$t_{ij}=j\cdot \frac{i}{f_{ri}},(j=1,2,\cdots ,m_{i})$$

(14) $$t_{rij}=j\cdot \frac{i}{RF_{i}},(j=1,2,\cdots ,p_{i})$$

Determine the interpolation algorithm. When the resampling points calculated in the previous step fall between the original two discrete data points, the values of the multi-sampling data points need to be interpolated. Commonly used interpolation methods are linear interpolation, Newton interpolation and Lagrange interpolation. In order to improve the calculation efficiency, this article uses linear interpolation method. The formula is as follows:

(15) $$x_{rip}=x_{ij}+\frac{x_{i(j+1)}-x_{ij}}{t_{i(j+1)}-t_{ij}}(t_{rip}-t_{ij})$$

where $x_{rip}$ is the *p*-th resampling point of fragment $x_{i}$; $t_{rip}$ is the resampling time of the *p*-th resampling point; $x_{ij}$, $x_{i\left(j+1\right)}$ are the *j*-th and (*j* + 1)-th data of the $x_{i}$ respectively; $t_{ij}$, $t_{i\left(j+1\right)}$ are the time label of *j*-th and (*j* + 1)-th data of the $x_{i}$ respectively.

After acquiring the resampled data points $x_{rip}$ of the monitoring data $x_{i}$ at all speeds, arrange the discrete data points in chronological order, and the resulting data sequence is the resampled data $x_{ri}$.

#### 2.3.3. The Improved One-Dimensional Convolutional Neural Network

The convolution networks used in the field of fault diagnosis are mostly based on the two-dimensional convolutional neural network structures. In our previous research [2], we introduced the one-dimensional convolutional neural network into the field of bearing fault diagnosis. Based on the previous research, this paper optimizes and tailors the network structure to a certain extent. To obtain the network with better performance, several experiments with different networks were conducted. The training dataset was the vibration signal collected under 1020 RPM and the testing dataset was the resampling signal with 1140 RPM. The diagnosis results of each network are shown in Table 1.

**Figure 4:**
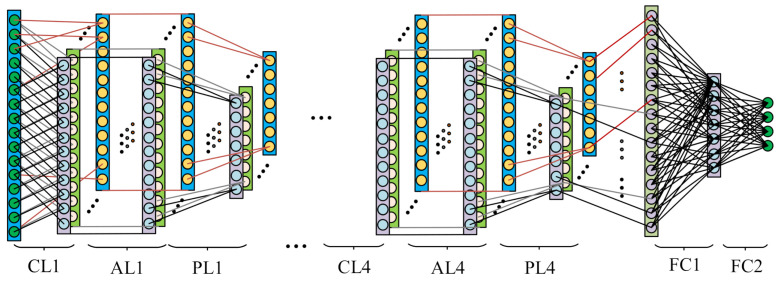
The improved one-dimensional convolutional neural network.

From the above table, we can find that the network with four convolutional layers and two fully connected layers achieves the highest accuracy. Additionally, the results show that the convolutional layers play a more important role than the fully connected layers. The networks with the same number of the convolutional layers tend to have the similar results. Thus, a one-dimensional convolutional neural network with convolutional layers and two fully connected layers is adopted for the fault diagnosis tasks. The structure and parameters of the network are shown as Table 2 and Figure 4.

As shown in Figure 4, the improved one-dimensional convolutional neural network includes four convolutional layers, four pooling layers, four activation layers and two fully connected layers. The input layer of the first layer contains 2048 nodes. The length of the convolution kernel in the first convolution layer is 64, which is used to amplify the receptive field of the convolution kernel and obtain richer spectral features. There are 16 convolution kernels in the first convolutional layer, which act as the filters to deal with the input one-dimensional vibration data. The size of all the convolution kernels in the second, third and fourth convolutional layers is 3 × 1. These kernels are used to extract detailed features. All the activation layers use ReLU activation functions. The first fully connected layer contains 512 nodes, and there are 20 nodes in the second fully connected layer. The two fully connected layers are used to perform nonlinear mapping on the features extracted by the convolutional layers.

The structure of the proposed 1D-CNN is shown as Table 2 and Figure 4. When training the network, we adopt the cross-entropy between the target class probability distribution and the estimated soft-max output probability distribution as the loss function. Let *q*(*x*) denote the estimated distribution and *p*(*x*) denote the target distribution; the function of the cross-entropy is shown as:

(16) $$Loss=H(p(x),q(x))=-\sum_{x}p(x)\log q(x)$$

The stochastic gradient descent (SGD) method is used as the optimizer, the batch size is set to 40, the learning rate is set to 1.0 × 10^−6^ and the number of iterations is 1000. When training the network, first of all, the data enhancement method is applied in the training dataset. The training data and testing data used in the experiments were one-dimensional vibration signals. Under a single working condition, the length of the original monitoring signals for each health state was set to N, the length of each input data of the network was 2048 and we sampled the training data at intervals of 10 data points in the original vibration data sequence. We got (N-2048)/10 + 1 samples from each original vibration data sequence. Secondly, batch normalization operation is applied in each convolutional layer. Since the network we use is 1D-CNN, the BatchNorm1d function is adopted to do this. Finally, in the fully connected layers, we adopt dropout operation and the rate of dropout is set to 0.5.

## 3. Results

In this study, two different bearing fault datasets were used to verify the effectiveness of the proposed algorithm under variable speed conditions. Firstly, in order to verify the superiority of the proposed algorithm, we used Case Western Reserve Bearing Database [40] to conduct experiments. In order to further verify the superiority of the algorithm, we also carried out verification experiments on the experimental platform built in our laboratory. The results show that the proposed method can effectively improve the accuracy of the fault diagnosis at different speeds. The proposed algorithm can better realize the migration diagnosis task from training data collected at a single speed to the fault data collected at other speeds.

### 3.1. Experiment on the Case Western Reserve University Bearing Dataset

#### 3.1.1. Dataset Description

**Figure 5:**
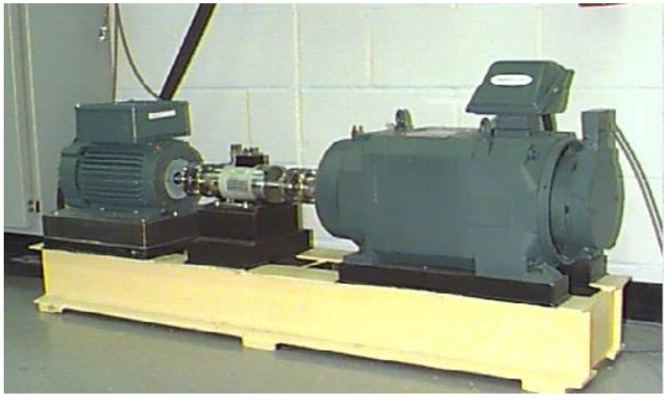
Motor driving mechanical system used by CWRU.

The Case Western Reserve University Bearing Data Center Website privates the ball bearing test data for normal and faulty bearings. All the acceleration data were collected in the experiments conducted using a 2 hp Reliance Electric motor. The vibration data were recorded for motor loads of 0 to 3 horsepower and the motor speeds of 1797 to 1730 RPM. The fault data were divided into four types: normal data, inner ring fault data, outer ring fault data and rolling element fault data. The device is shown as Figure 5.

In this study, we made four datasets, A, B, C and D, to conduct the experiment. In each dataset, there are training data and testing data. The training data in each dataset only contain the data of each health state collected under one speed. The testing data contain the data of each health state collected under four speeds. The comparative experiments directly used the deep convolutional neural network to verify the migration diagnosis effect between different rotation speed states, without using the order tracking algorithm to preprocess the original data. The descriptions of the bearing fault datasets are as shown in Table 3.

#### 3.1.2. Order Tracking Resample and Transfer Diagnosis

In each dataset, we used the speed of the training data as the basic rotation speed; then calculated the resampling frequency of the original vibration data under each speed; and finally resampled the original data according to the resample frequency of each speed. In each health state under each speed, we took 3000 samples and the length of each sample was 2048. In each dataset, the training dataset contains four types of health state data (health data, inner race fault data, outer race fault data and ball fault data) under one speed; thus, the total number of the training data is 3000 × 4 = 12,000. The testing dataset in each dataset contains the resampled data with all the types of health sate under each rotation speed; thus, the total number of testing data in each dataset was 12,000 and the length of each data sample was also 2048.

**Figure 6:**
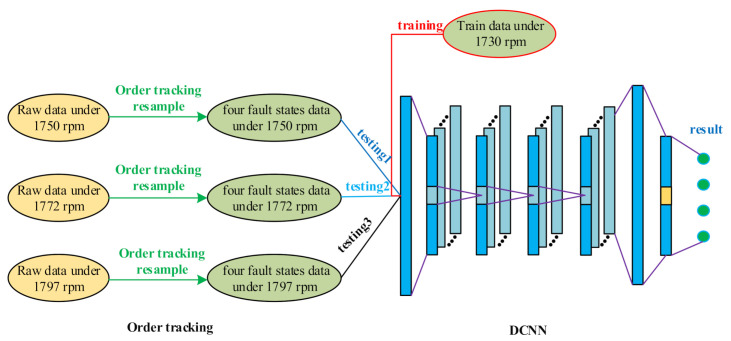
The data processing flow.

After getting all the training data and testing data in each dataset, we can carried out the experiments. Firstly, we trained the deep convolutional neural network with training data, the input the test data under each speed to the trained network and got the migration classification accuracy from the train data to the test data at 1730, 1750, 1772 and 1797 RPM respectively. The detailed data processing flow is as shown in Figure 6.

#### 3.1.3. Experiment Results

In order to prove the effectiveness of the order tracking algorithm, this study used Case Western Reserve University Bearing Data Set for experiments. Motor bearings were seeded with faults using electro-discharge machining (EDM). Faults ranging from 0.007 inches in diameter to 0.040 inches in diameter were introduced separately at the inner raceway, rolling element (i.e., ball) and outer raceway. Faulted bearings were reinstalled into the test motor and vibration data were recorded for motor loads of 0 to 3 horsepower (motor speeds of 1797 to 1720 RPM). In the above data, we selected health status data, inner ring fault data, outer ring fault data and ball fault data at four speeds for order tracking and resampling. The experimental results are shown in the following figure.

**Figure 7:**
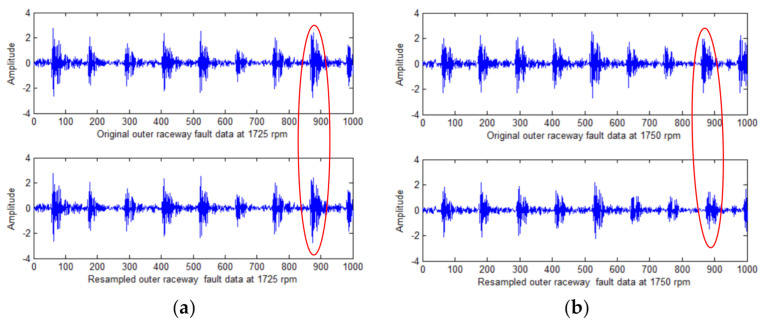

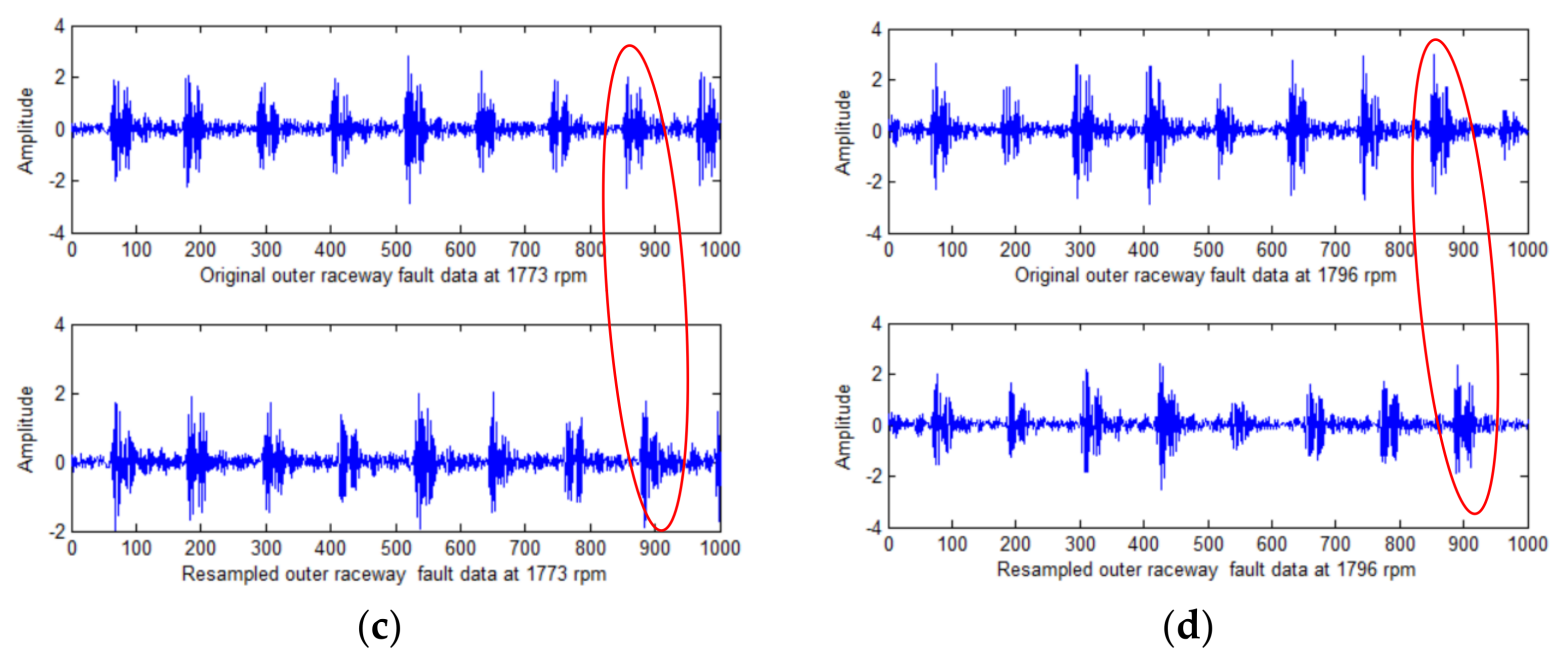
The experimental results of order tracking: (**a**) resampling results at 1725 speed; (**b**) resampling results at 1750 speed; (**c**) resampling results at 1773 speed; (**d**) resampling results at 1796 speed.

Figure 7a is the data resampling effect at 1725 RPM, 1725 RPM is the basic RPM, its sampling frequency is the reference sampling frequency and the resampled data are the same as the original data. Figure 7b–d shows the data resampling effects at 1750, 1773 and 1796 RPM respectively. The sampling data at these three RPMs have a better effect of restoring the original data waveform. The processing effect of the tracking resampling algorithm is visually manifested as an axial scaling of the original data on the time axis.

**Figure 8:**
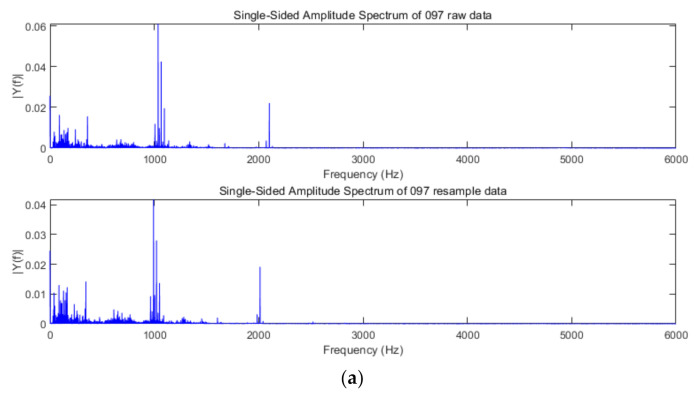

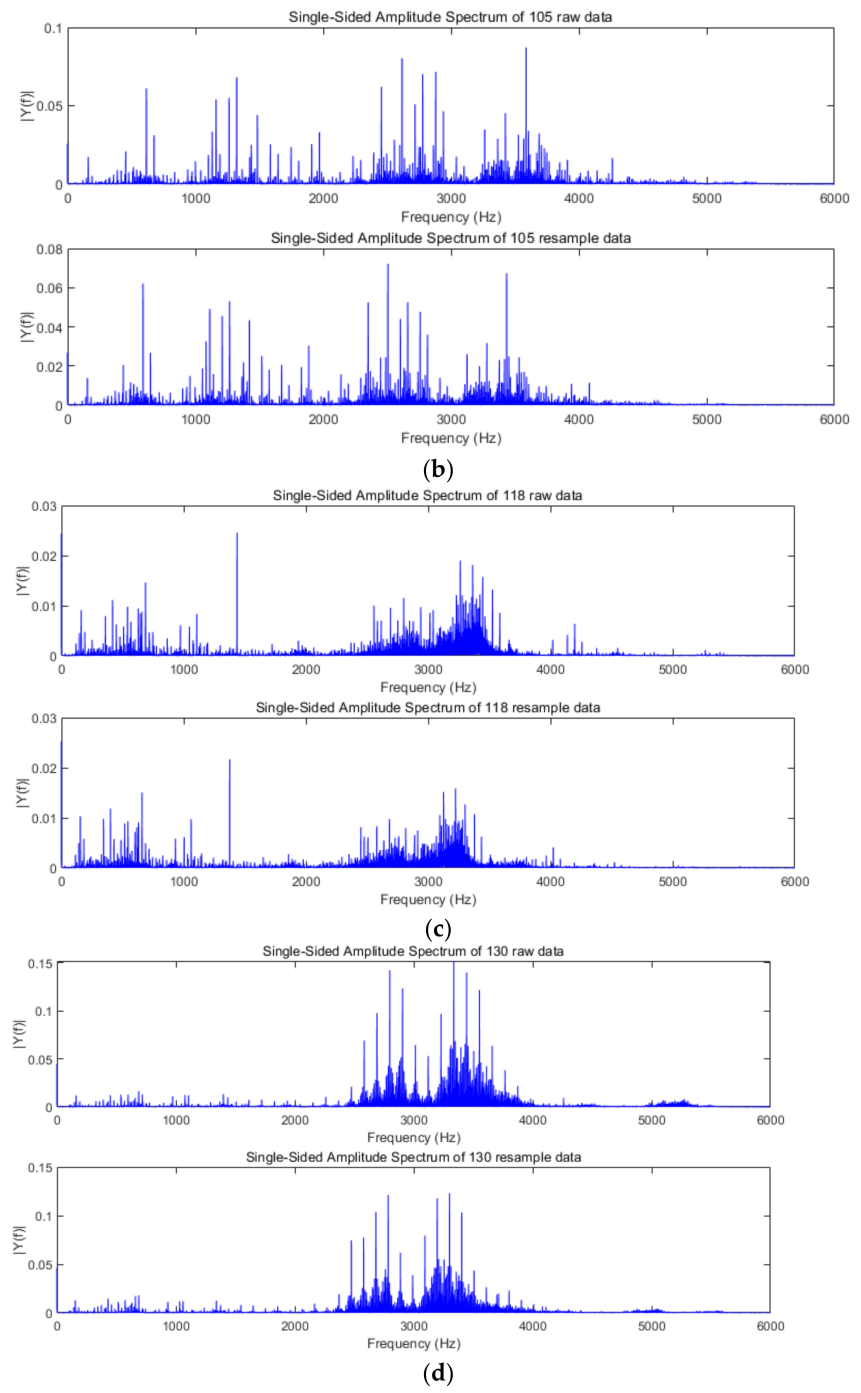
The spectral characteristics of raw data and resampling data. (**a**) Spectrum of vibration data in healthy state; (**b**) spectrum of vibration data in inner race fault state; (**c**) spectrum of vibration data in ball fault state; (**d**) spectrum of vibration data in outer race fault state.

The purpose of order tracking is to overcome the ambiguity of the fault characteristic spectrum, which is caused by the speed change. We set 1730 rpm as the base speed, and the corresponding vibration data at the 1730 speed as the basis sampling frequency. The order tracking algorithm resampled the fault data at 1797 speed, and the Fourier transform was performed on the original data before resampling and the fault data after resampling. The results of Fourier transform are shown as Figure 8.

**Figure 9:**
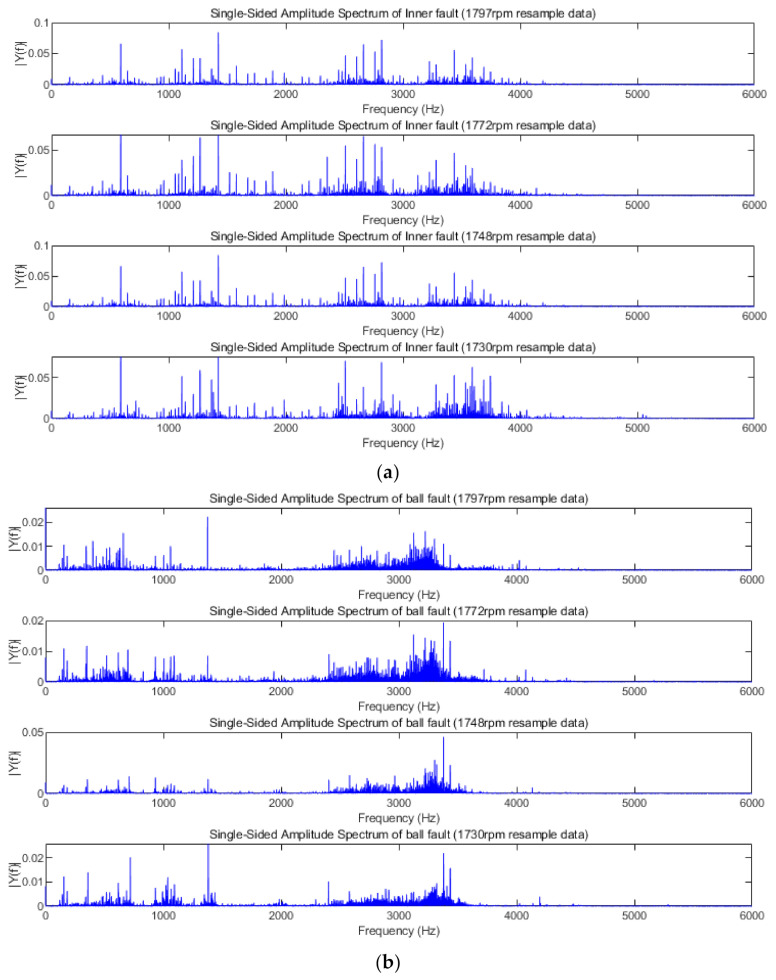

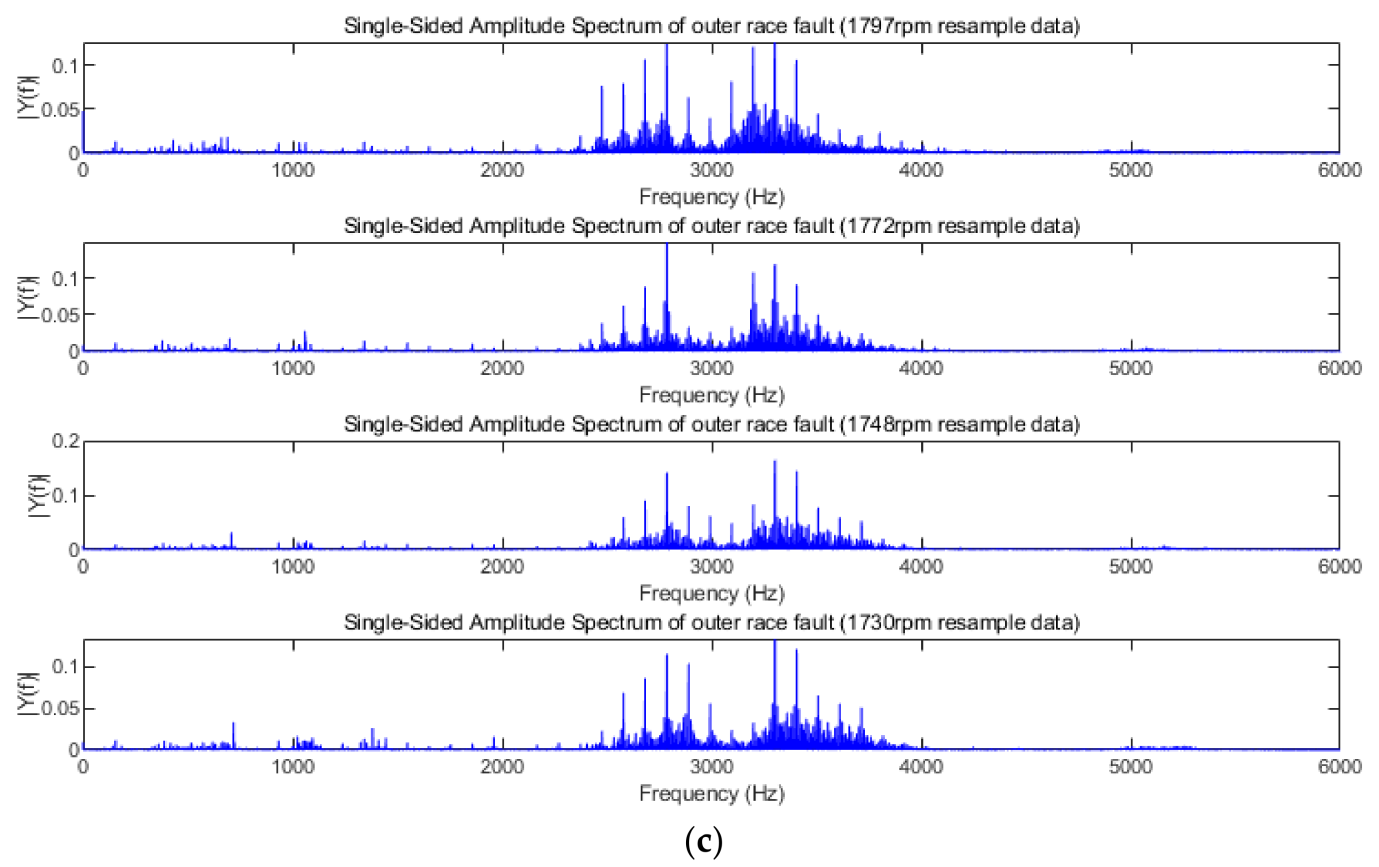
The spectral characteristics of resampling data at each speed. (**a**) Spectrum of inner race fault resampling data at each speed; (**b**) spectrum of ball fault resampling data at each speed; (**c**) spectrum of outer race fault resampling data at each speed.

In Figure 8, it can be found that the spectral characteristics of the data change after resampling, and the characteristic frequency of each order becomes smaller. These results are consistent with the expected effects of the order tracking algorithm. After resampling the original fault data with the order tracking algorithm, the spectrum characteristics of the same type of fault data obtained at different speeds are consistent, as is shown in Figure 9. This proves the effectiveness of the adopted order tracking algorithm in processing fault data under variable speed conditions.

**Figure 10:**
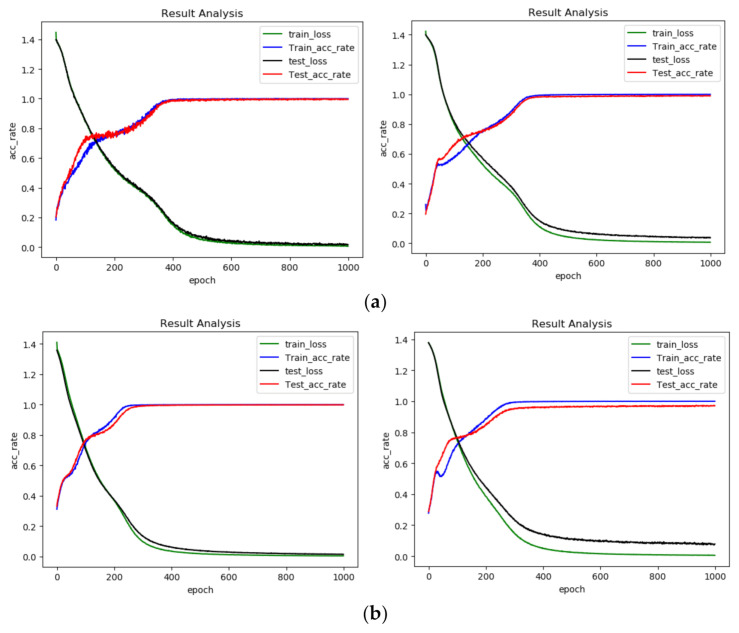

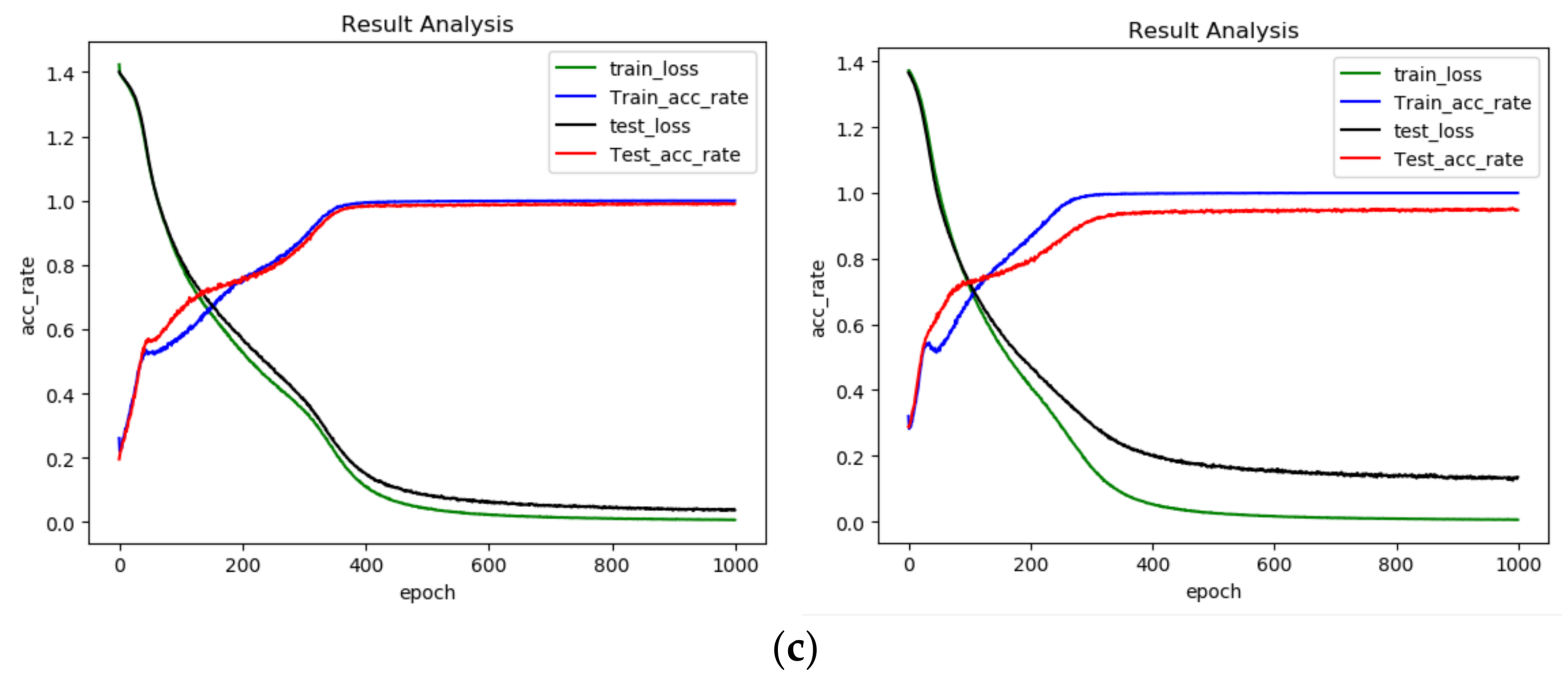
Diagnosis results of each dataset. (**a**) Diagnosis results of dataset A (the former is OT-1DCNN, the latter is DCNN); (**b**) diagnosis results of dataset B (the former is OT-1DCNN, the latter is DCNN); (**c**) diagnosis results of dataset C (the former is OT-1DCNN, the latter is DCNN).

In dataset A, we took the collected data with 1730 rpm as the training data to train the DCNN. With the trained network, we classified the resampled fault data with 1730, 1750, 1772 and 1797 rpm. The classification accuracy of the network’s fault data under each speed state can reflect the proposed algorithm’s ability to diagnose fault data under variable speed conditions. The experiments were implemented using the Pytorch framework in Pycharm Community 2019.1. The classification results with each dataset are shown as Figure 10 and Table 4.

From Table 4 and Figure 10, we can find that the results got from OT-1DCNN are better than those of DCNN. On dataset A, the average accuracy of OT-1DCNN was 99.69%, which is higher than 98.93% by 0.74%. On dataset B, the average accuracy of OT-1DCNN was 99.76% and the accuracy of DCNN was 97.14%; the former is higher than the later by 2.62%. On dataset C, the improvement of the proposed algorithm was the most obvious. The average accuracies of OT-1DCNN and DCNN were 98.95% and 94.92% respectively, which means the improvement rate is 4.03%. In this experiment, we can find that the improvement rate is higher when the speed difference between source domain data and the target domain is larger.

**Figure 11:**
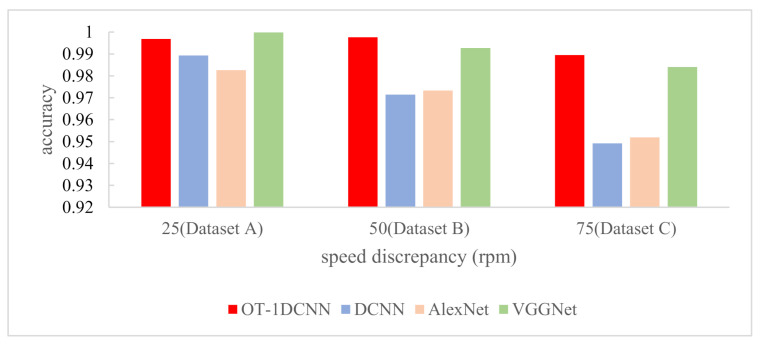
The results of comparative experiments with CWRU data.

To verify the superiority of OT-1DCNN, we conducted the comparative experiments with CWRU data. In the comparative experiments, the OT-1DCNN, DCNN, AlexNet and VGGNet were adopted on dataset A, dataset B and dataset C respectively. In order to keep the consistency of the input data, we modified the structures of the DCNN, AlexNet and VGGNet, making them suitable for processing the one-dimensional vibration signals. The number of the four networks’ input layer’s nodes was 2048, which is the same as OT-1DCNN. Among them, DCNN contained 4 convolutional layers and 2 fully connected layers; AlexNet contained 5 convolutional layers and 3 fully connected layers; VGGNet contained 6 convolutional layers and 2 fully connected layers. The results of comparative experiments are shown in Figure 11 and Table 5.

With dataset A, the accuracies of OT-1DCNN, DCNN, AlexNet and VGGNet were 0.9969, 0.9893, 0.9826 and 0.9998 respectively. VGGNet got the highest accuracy, which was higher than OT-1DCNN by just 0.29%. With dataset B, the accuracies were 0.9976, 0.9714, 0.9733 and 0.9927 respectively. With dataset C, the accuracies were 0.9895, 0.9492, 0.9519 and 0.9841 respectively. With dataset B and dataset C, OT-1DCNN got the highest accuracy.

### 3.2. Experiment on Our Own Dataset

#### 3.2.1. Dataset Description

**Figure 12:**
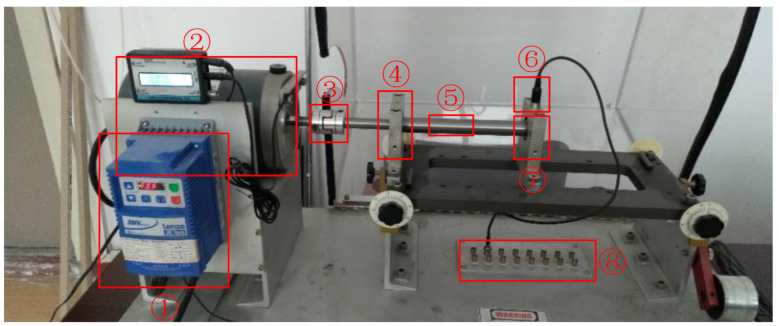
The self-made bearing fault platform. ① motor driver, ② three-phase AC motor, ③ coupler, ④ healthy bearing and its foundation, ⑤ rotating shaft, ⑥ acceleration sensor, ⑦ fault bearing and its foundation, ⑧ signal collection device.

To further verify the effectiveness of the proposed algorithm, we carried out further experiments on our own bearing fault platform. The self-made device is shown as Figure 12. The test device uses a deep groove ball bearing for testing, whose model is 6204. The health status of bearing includes health status, inner ring failure and outer ring failure. The fault area is formed by EDM. The experimental dataset contains data collected under four speed states: 900, 1020, 1140 and 1260 RPM, and the sampling frequency is 25.6 kHz.

On this experimental platform, we only used the fault data collected at 900 RPM as the training dataset, and the fault data collected at 1020, 1140 and 1260 RPM as the test datasets. The fault data under each speed state included three states: health state, inner ring fault and outer ring fault. There were 3000 samples in each state of health in each speed state, and each sample contained 2048 vibration data points. The datasets used in the test were as shown in Table 6.

#### 3.2.2. Experiment Results

**Figure 13:**
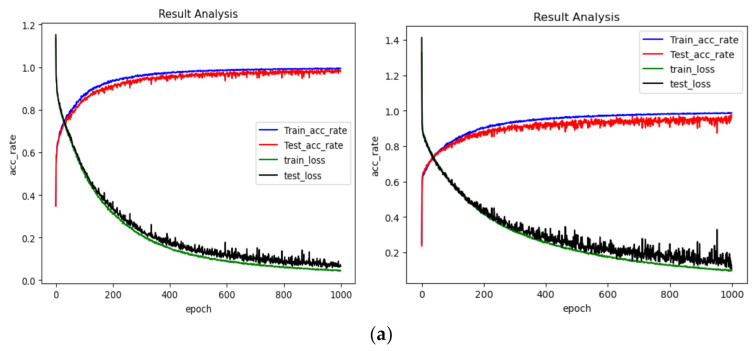

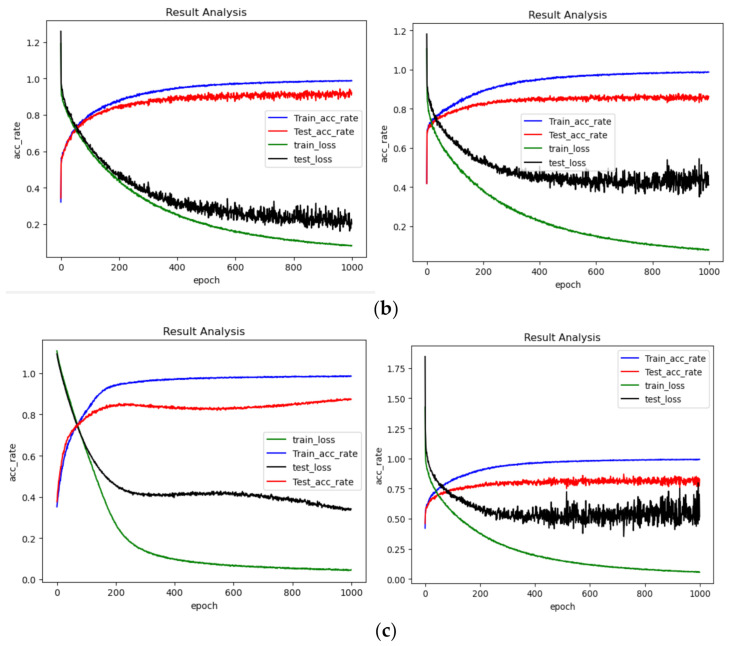
Diagnosis results of each dataset from self-made device: (**a**) diagnosis results of dataset A (the former is OT-1DCNN, the latter is DCNN); (**b**) diagnosis results of dataset B (the former is OT-1DCNN, the latter is DCNN); (**c**) diagnosis results of dataset C (the former is OT-1DCNN, the latter is DCNN).

In this experiment, we used the same data processing process as the fault data on the Case Western Reserve bearing database. For the fault data set containing only three types, we adjusted the number of neurons in the last layer to three. The results of the experiment are shown as Figure 13 and Table 7.

From Table 7 and Figure 13, we can find that the results gotten from OT-1DCNN are better than those of DCNN. On dataset A, the average accuracy of OT-DCNN was 98.40%, which is higher than the 95.19% of DCNN by 3.21%. On dataset B, the average accuracy of OT-DCNN was 92.69% and the accuracy of DCNN was 85.42%; the former is higher than the later by 7.27%. On dataset C, the improvement of the proposed algorithm was the most obvious. The average accuracies of OT-1DCNN and DCNN were 87.35% and 76.35% respectively, for which the improvement rate is 11.00%. In this experiment, we can find that the improvement rate is higher when the speed difference between source domain data and the target domain is larger.

To further verify the superiority of OT-1DCNN, we conducted further experiments based on the data collected on the self-made test device. Compared with CWRU dataset, the speed difference between the training dataset and the testing dataset is greater. This can more clearly prove the migration diagnosis ability of the OT-1DCNN. The structures of the four networks used in this experiment were exactly the same as those in experiment A. The results of comparative experiments with the self-made dataset are shown as Table 8.

**Figure 14:**
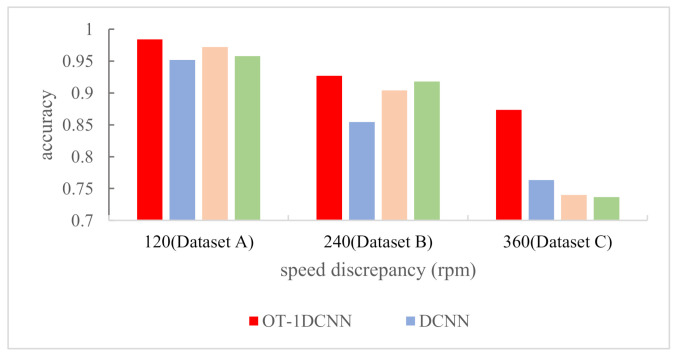
The results of comparative experiments with self-made dataset.

From Table 8 and Figure 14, we can see that with dataset A, the accuracies of OT-1DCNN, DCNN, AlexNet and VGGNet were 0.9840, 0.9519, 0.9722 and 0.9578 respectively. With dataset B, the accuracies were 0.9269, 0.8542, 0.9041 and 0.9180 respectively. With dataset C, the accuracies were 0.8735, 0.7635, 0.402 and 0.7366 respectively. With all three datasets, OT-1DCNN got the highest accuracy.

## 4. Discussion

The proposed OT-1DCNN method can effectively deal with the problem of decreased fault diagnosis accuracy, which is caused by variable speed conditions. The proposed algorithm firstly resamples the original signals with order tracking algorithm. Then a one-dimensional convolutional neural network is used to adaptively extract the features of the fault signals and classify the data automatically. In the network training stage, only the fault data obtained at one speed is adopted to train the network. The trained network performed well in dealing with the fault data collected at other speeds, even though the testing data had a different distribution from the training data. Our results in the experiments show the OT-1DCNN algorithm performs better than the comparative methods.

In the proposed OT-1DCNN algorithm, it was proven in the experiments that the order tracking algorithm can improve the stability of the frequency spectrum distribution of the fault characteristic signal in variable speeds condition. It can transform the vibration signal from time domain to angle domain by resampling the original signals. For the resampling signals, the spectrum characteristics will not change along with the variable speeds. In this case, the problem of an unstable signal spectrum will be overcome. Additionally, the convolutional neural network has the ability to adaptively extract the effective information hidden in the original monitoring signal, avoiding the complex process of feature design and the extraction.

From the perspective of math principle, the proposed OT-1DCNN algorithm can completely solve the problem of decreased diagnostic accuracy of the network, which is caused by variable speeds. However, when the discrepancy of speeds between training data and testing data gets bigger, the accuracy of the network still decreases to a certain extent. The authors will investigate these issues in the future works.

## 5. Conclusions

This paper proposes a two-stage bearing fault diagnosis algorithm to realize the diagnosis of bearing fault data under different speed conditions. The algorithm effectively combines the order tracking algorithm with the one-dimensional convolutional neural network. The order tracking algorithm is good at dealing with the problem of an unstable signal spectrum in variable speed conditions. The one-dimensional convolutional neural network can adaptively extract the features of the fault signals and classify the data automatically. The proposed OT-1DCNN algorithm adopts these methods at different data processing stages to make full use of the advantages of them. By doing this, the proposed method can reduce the dependence on the completeness of the training datasets. The algorithm only uses the fault data collected under one speed as the training dataset, and realizes the migration diagnosis of the fault data collected at other speed states. This study used the Case Western Reserve bearing database and our own bearing failure data experimental platform to obtain experimental data for experimental verification. In the condition with the largest difference in speed with each dataset, the accuracy of the proposed method was higher than for the comparative methods by 0.54% with CWRU dataset and by 11.00% with our own dataset respectively. The experimental results show that the proposed algorithm can significantly improve the DCNN migration diagnosis effect under variable speed conditions in the time domain with the same training datasets. In the experiment, we found that the migration diagnosis accuracy of the network will decrease as the speed difference between the training set and the test set increases. Future research work will continue to explore the reasons for the decline in network classification accuracy.

## Figures and Tables

**Table 1:** The diagnosis results with different networks.

Number of Convolutional Layers/Number of Fully Connected Layers	2/2	3/2	4/2	5/2	2/3	3/3	4/3	5/3
Test 1	0.8526	0.9533	0.9817	0.981	0.8633	0.9468	0.9785	0.9653
Test 2	0.8066	0.9683	0.9782	0.9853	0.7966	0.9568	0.9651	0.9647
Test 3	0.8735	0.9421	0.98	0.9421	0.8366	0.9548	0.9836	0.9651
Test 4	0.7566	0.9255	0.9848	0.9735	0.7658	0.9647	0.9825	0.9685
Test 5	0.8218	0.9784	0.9833	0.9566	0.8265	0.9452	0.9627	0.9647
Test 6	0.8355	0.9524	0.9852	0.9894	0.8467	0.9687	0.9534	0.9643
Test 7	0.8647	0.9486	0.984	0.9833	0.8672	0.9568	0.9438	0.9681
Test 8	0.8533	0.9548	0.9846	0.9866	0.8642	0.9533	0.9569	0.9584
Test 9	0.8436	0.9523	0.9828	0.988	0.8586	0.9642	0.9541	0.9732
test 10	0.8211	0.9366	0.9857	0.9856	0.8464	0.9583	0.9762	0.9679
Max	0.8735	0.9784	0.9857	0.9894	0.8672	0.9687	0.9836	0.9732
Min	0.7566	0.9255	0.9782	0.9421	0.7658	0.9452	0.9438	0.9584
Average	0.8342	0.9529	0.9827	0.9762	0.8362	0.9568	0.9645	0.9658

**Table 2:** Details of DCNN model used in experiments.

No.	Layer Type	Kernel Size/Stride	Kernel Number	Output Size (Width × Depth)	Padding
1	Convolution1	64 × 1/8 × 1	16	256 × 16	Yes
2	Pooling1	2 × 1/2 × 1	16	128 × 16	No
3	Convolution2	3 × 1/1 × 1	32	128 × 32	Yes
4	Pooling2	2 × 1/2 × 1	32	64 × 32	No
5	Convolution3	3 × 1/1 × 1	64	64 × 64	Yes
6	Pooling3	2 × 1/2 × 1	64	32 × 64	No
7	Convolution4	3 × 1/1 × 1	64	32 × 64	Yes
8	Pooling4	2 × 1/2 × 1	64	16 × 64	No
9	Fully-connected	512	1	512 × 1	
10	Fully-connected	20	1	20 × 1	
11	Softmax	4	1	4	

**Table 3:** The datasets used in experiment A.

Fault Location		Normal	Inner Race	Outer Race	Ball	Speed
Category Labels		1	2	3	4	
Dataset A	Training data	3000	3000	3000	3000	1730
	Testing data	3000	3000	3000	3000	1750
Dataset B	Training data	3000	3000	3000	3000	1730
	Testing data	3000	3000	3000	3000	1772
Dataset C	Training data	3000	3000	3000	3000	1730
	Testing data	3000	3000	3000	3000	1797

**Table 4:** Diagnosis results of each dataset.

Dataset	A		B		C	
	OT-1DCNN	DCNN	OT-1DCNN	DCNN	OT-1DCNN	DCNN
Test 1	0.9975	0.9885	0.9933	0.9703	0.9885	0.9477
Test 2	0.9950	0.9895	0.9975	0.9717	0.9895	0.9480
Test 3	0.9975	0.9888	0.9958	0.9733	0.9888	0.9483
Test 4	0.9958	0.9888	0.9975	0.9724	0.9905	0.9498
Test 5	0.9950	0.9905	0.9992	0.9730	0.9894	0.9475
Test 6	0.9983	0.9894	0.9950	0.9716	0.9898	0.9484
Test 7	0.9967	0.9898	0.9992	0.9699	0.9895	0.9487
Test 8	1.0000	0.9895	1.0000	0.9700	0.9891	0.9534
Test 9	0.9967	0.9891	0.9992	0.9707	0.9893	0.9526
Test 10	0.9963	0.9893	0.9992	0.9708	0.9905	0.9479
Max	1.0000	0.9905	1.0000	0.9733	0.9905	0.9534
Min	0.9950	0.9885	0.9992	0.9699	0.9885	0.9475
Average	0.9969	0.9893	0.9976	0.9714	0.9895	0.9492

**Table 5:** The results of comparative experiments with CWRU data.

Algorithms	Dataset A	Dataset B	Dataset C
OT-1DCNN	0.9969	0.9976	0.9895
DCNN	0.9893	0.9714	0.9492
AlexNet	0.9826	0.9733	0.9519
VGGNet	0.9998	0.9927	0.9841

**Table 6:** The datasets used in the experiment B.

Fault Location		Normal	Inner Race	Outer Race	Speed
Category Labels		1	2	3	
Dataset A	Training data	3000	3000	3000	900
	Testing data	3000	3000	3000	1020
Dataset B	Training data	3000	3000	3000	900
	Testing data	3000	3000	3000	1140
Dataset C	Training data	3000	3000	3000	900
	Testing data	3000	3000	3000	1260

**Table 7:** Diagnosis results of each dataset from the self-made device.

Dataset	A		B		C	
	OT-1DCNN	DCNN	OT-1DCNN	DCNN	OT-1DCNN	DCNN
Test 1	0.9817	0.9558	0.9291	0.8508	0.8734	0.7878
Test 2	0.9882	0.9587	0.9127	0.8635	0.8727	0.7504
Test 3	0.9800	0.9659	0.9331	0.8510	0.8759	0.7424
Test 4	0.9848	0.9472	0.9218	0.8644	0.8736	0.7698
Test 5	0.9833	0.9421	0.9261	0.8448	0.8726	0.7568
Test 6	0.9852	0.9603	0.9282	0.8613	0.8721	0.7699
Test 7	0.9840	0.9294	0.9422	0.8500	0.8730	0.7709
Test 8	0.9846	0.9589	0.9294	0.8521	0.8766	0.7693
Test 9	0.9828	0.9580	0.9235	0.8570	0.8711	0.7608
Test 10	0.9857	0.9422	0.9227	0.8466	0.8741	0.7572
Max	0.9882	0.9659	0.9422	0.8644	0.8766	0.7878
Min	0.9828	0.9294	0.9127	0.8448	0.8711	0.7424
Average	0.9840	0.9519	0.9269	0.8542	0.8735	0.7635

**Table 8:** The results of comparative experiments with the self-made dataset.

Algorithms	Dataset A	Dataset B	Dataset C
OT-1DCNN	0.9840	0.9269	0.8735
DCNN	0.9519	0.8542	0.7635
AlexNet	0.9722	0.9041	0.7402
VGGNet	0.9578	0.9180	0.7366
